# Atypical chemokine receptor ACKR2 controls branching morphogenesis in the developing mammary gland

**DOI:** 10.1242/dev.139733

**Published:** 2017-01-01

**Authors:** Gillian J. Wilson, Kay D. Hewit, Kenneth J. Pallas, Claire J. Cairney, Kit M. Lee, Christopher A. Hansell, Torsten Stein, Gerard J. Graham

**Affiliations:** 1 Chemokine Research Group, Institute of Infection, Immunity and Inflammation, College of Medical, Veterinary and Life Sciences, University of Glasgow, Glasgow G12 8TT, UK; 2 Institute of Cancer Sciences, University of Glasgow, Glasgow G61 1QH, UK

**Keywords:** ACKR2, Mammary gland, Chemokines, Mouse, Branching

## Abstract

Macrophages are important regulators of branching morphogenesis during development and postnatally in the mammary gland. Regulation of macrophage dynamics during these processes can therefore have a profound impact on development. We demonstrate here that the developing mammary gland expresses high levels of inflammatory CC-chemokines, which are essential *in vivo* regulators of macrophage migration. We further demonstrate that the atypical chemokine receptor ACKR2, which scavenges inflammatory CC-chemokines, is differentially expressed during mammary gland development. We have previously shown that ACKR2 regulates macrophage dynamics during lymphatic vessel development. Here, we extend these observations to reveal a novel role for ACKR2 in regulating the postnatal development of the mammary gland. Specifically, we show that *Ackr2*^−/−^ mice display precocious mammary gland development. This is associated with increased macrophage recruitment to the developing gland and increased density of the ductal epithelial network. These data demonstrate that ACKR2 is an important regulator of branching morphogenesis in diverse biological contexts and provide the first evidence of a role for chemokines and their receptors in postnatal development processes.

## Abstract (precis)

**Summary:** *Ackr2*^−/−^ mice display precocious mammary gland development as a result of impaired chemokine scavenging and increased macrophage recruitment to the mammary gland.

## INTRODUCTION

During embryonic development, an epithelial placode forms in the murine mammary gland mid-gestation (E11.5) and at birth a rudimentary structure is present (Hens and Wysolmerski, 2005). Most organs are patterned during embryogenesis or in the first week after birth (Howlin et al., 2006; Wiseman and Werb, 2002). However, the mammary gland is unique, as continual postnatal development occurs throughout the female reproductive lifetime. Ovarian hormones begin to be released at puberty (around 3 weeks) and terminal end buds (TEBs) form at the distal tip of the epithelial ducts. TEBs proliferate, grow invasively through the fat pad and branch by bifurcation until the limit of the fat pad is reached and they regress (Wiseman and Werb, 2002). During pregnancy, ductal structures branch further and differentiate into lobuloalveoli to produce milk during lactation. After weaning, involution occurs, where 90% of the epithelium undergoes programmed cell death to allow the gland to return to its pre-pregnancy state (Wiseman and Werb, 2002).

The mammary gland fat pad comprises mainly fibroblasts, pre-adipocytes, adipocytes and immune cells, and is separated from the epithelial network by a periductal stroma of fibroblasts and extracellular matrix (ECM) (Parmar and Cunha, 2004). Macrophages, mast cells and eosinophils surround the growing TEBs (Gouon-Evans et al., 2000; Lilla and Werb, 2010). Macrophages are found in all tissues within the body and in addition to mounting immune responses during inflammation, their phagocytic and cytokine-producing properties are required for many tissue remodelling processes during development. Colony stimulating factor 1 (CSF1) is the main growth factor for macrophages, and *Csf1*-deficient mice (*Csfm^op^/Csfm^op^*) are impaired in their ability to form TEBs, show reduced ductal elongation and branching during puberty (Gouon-Evans et al., 2000; Pollard and Hennighausen, 1994), and have impaired development of lobuloalveoli in pregnancy. Macrophages are found mainly around the neck or within the TEBs, produce proteinases and growth factors, and promote collagen fibrillogenesis (Gouon-Evans et al., 2000; Ingman et al., 2006). Eosinophils and mast cells are also required for mammary gland development, as CCL11-null and mast cell-deficient mice, respectively, have impaired TEB and branch formation (Gouon-Evans et al., 2000; Lilla and Werb, 2010).

Although the role of CCL11 in recruiting eosinophils to the mammary gland has been well defined (Gouon-Evans et al., 2000), there is limited evidence regarding the involvement of other chemokines and their receptors in mammary gland development. Chemokines are chemotactic cytokines that control cell migration and have multiple functions in inflammation and homeostasis (Griffith et al., 2014; Zlotnik and Yoshie, 2000), including the regulation of macrophage dynamics during tissue remodelling. ACKR2 is an example of a non-signalling, 7-transmembrane spanning atypical chemokine receptor that lacks the DRYLAIV motif characteristic of conventional chemokine receptors (Bachelerie et al., 2014; Nibbs and Graham, 2013). ACKR2 is expressed by lymphatic endothelial cells, by some leukocyte populations, including B1 cells, and by stromal cells during inflammation (Hansell et al., 2011; McKimmie et al., 2008; Nibbs et al., 2001; Singh et al., 2012). ACKR2 is required for resolution of the inflammatory response, by internalising CC-chemokines and depositing them in the lysosome for degradation (Fra et al., 2003; Weber et al., 2004). Exaggerated inflammation is seen at all sites of normal ACKR2 expression in *Ackr2*^−/−^ mice (Di Liberto et al., 2008; Jamieson et al., 2005; Martinez de la Torre et al., 2007; Nibbs et al., 2007).

Recently, we demonstrated that ACKR2 is an essential regulator of dermal macrophage dynamics during embryonic branching morphogenesis. Compared with wild-type mice, *Ackr2*^−/−^ mice have increased lymphatic vessel density that is associated with altered recruitment and proximity of pro-lymphangiogenic macrophages to developing lymphatic vessels at a range of tissue sites (Lee et al., 2014). Here, we show that ACKR2 has a broader role in the regulation of macrophage dynamics during branching morphogenesis. We demonstrate strong expression of inflammatory CC-chemokine ligands for ACKR2 during mammary gland development and provide evidence that ACKR2 is expressed by stromal fibroblasts in the developing mammary gland. Analysis of developing mammary gland structures reveals that ACKR2 regulates epithelial branching by restricting levels of macrophage-attracting chemokines. In *Ackr2*^−/−^ mice, levels of these attractants are elevated, leading to increased recruitment of macrophages and a precocious mammary gland developmental phenotype. This study therefore sheds important light on the regulation of macrophage dynamics during mammary gland development and identifies ACKR2 as a crucial player in this process. This study also provides the first evidence of chemokine receptor involvement in postnatal development.

## RESULTS

### Chemokines and their receptors are abundantly expressed during mammary gland development

**Fig. 1.**
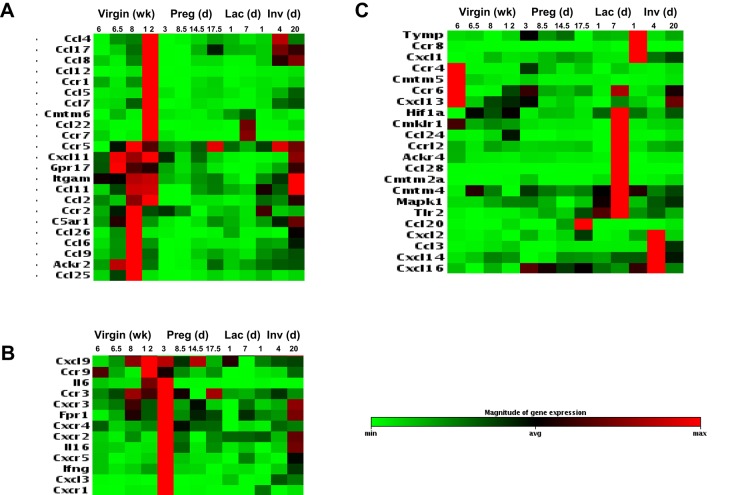
**Transcriptional regulation of chemokines and their receptors during mammary gland development.** Heat-map representation of chemokine transcript levels in the developing mammary gland showing markedly different patterns of chemokine and chemokine receptor expression. For ease of interpretation, the data have been subdivided according to chemokine expression patterns common to (A) virgin development (*n*=3), (B) pregnancy (*n*=2), and (C) lactation and involution (*n*=2).

Using qRT-PCR-based arrays, we investigated the expression of chemokines, their receptors and related molecules in the mammary gland during development, pregnancy, lactation and involution. Heat-map visualisation of the data is shown in Fig. 1, which reveals markedly different patterns of chemokine and chemokine receptor expression in the mammary gland in the four contexts studied. Specifically, our data demonstrate that inflammatory CC-chemokines (CCL2, CCL4-CCL9, CCL11, CCL17, CCL22 and CCL26) and their receptors (CCR1, CCR2, CCR5) are strongly expressed at different stages in the development of the mammary gland in virgin mice (Fig. 1A). The strongest expression of the CC-chemokines and their receptors was observed in mice aged between 6.5 and 12 weeks. The data also show that ACKR2 is expressed in the developing mammary gland in mice aged between 6.5 and 8 weeks. In contrast to mammary gland development, inflammatory CXC-chemokine activity is strongly apparent in the remodelling mammary gland during pregnancy (Fig. 1B). Notably, there is marked upregulation of CXCR1, CXCR2 and CXCR3 within 3 days of conception. In addition, the non-inflammatory CXC-chemokine receptors CXCR4 and CXCR5 are also expressed at this time point. Therefore CXC-chemokine activity typifies the early stages of the mammary gland response to pregnancy. The expression data from lactating and involuting mammary glands revealed consistent patterns but these did not have the clear chemokine-subfamily associations that are observed with developing and pregnant mammary glands (Fig. 1C). Thus, these data demonstrate strong expression of inflammatory CC-chemokines and their scavenger receptor ACKR2 during mammary gland development.

### ACKR2 controls mammary gland branching morphogenesis during development

**Fig. 2.**
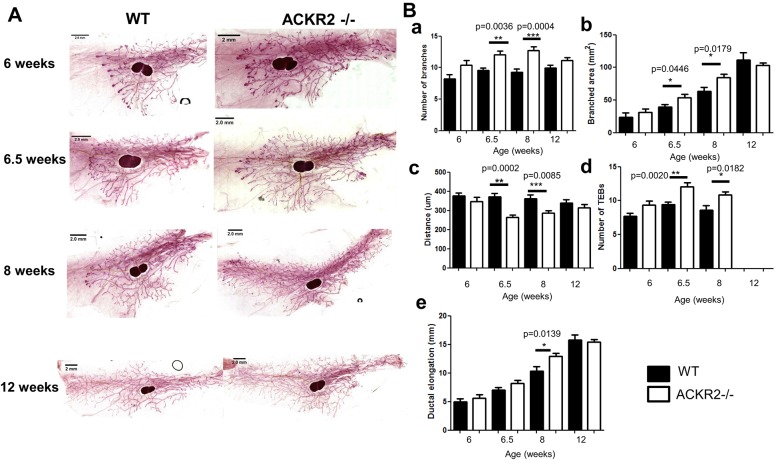
**ACKR2 controls** **branching morphogenesis during development.** (A) Representative Carmine Alum whole mounts prepared from virgin wild-type and *Ackr2^−/−^* mammary glands at 6 (*n*=6), 6.5 (*n*=8), 8 (*n*=8) and 12 weeks (*n*=9) are shown. Scale bars: 2 mm. (B) Branching morphogenesis was quantified by measuring: (a) the number of branches where each data point represents the average from three individual fields of view (FOV) (5×) per gland; (b) the area of branching from the inguinal lymph node using ImageJ; (c) the distance between branches where each data point represents the average from three measurements taken from each of three individual FOV (5×) per gland. (d) The number of TEBs was determined as the average number from three FOV (5×). (e) Ductal elongation was measured from the middle of the inguinal lymph node to the furthest edge of ductal outgrowth using ImageJ. Error bars represent s.e.m. Significantly different results are indicated.

Branching morphogenesis is an important process in the mammary gland, generating the ductal epithelial network throughout postnatal development (Macias and Hinck, 2012). Macrophages are known to contribute to this process (Gouon-Evans et al., 2000; Wynn et al., 2013), and their migration and dynamics in this context will be regulated by a number of the inflammatory chemokines identified in Fig. 1A. These chemokines are also ligands for ACKR2 and thus we next determined whether ACKR2 contributes to the regulation of branching morphogenesis in the mammary gland, as it does in embryonic skin (Lee et al., 2014). Carmine Alum whole-mount staining of the fourth inguinal mammary gland was carried out on age- and weight-matched virgin wild-type and *Ackr2*^−/−^ mice (Fig. 2A). Defined developmental time points were chosen to represent puberty (6 and 6.5 weeks) and adulthood (8 and 12 weeks), and at each time point mammary gland development was quantified (Fig. 2B). At 6 weeks, ACKR2 deficiency does not affect branching. However at 6.5 and 8 weeks old, *Ackr2*^−/−^ mice show accelerated development, as evidenced by an increased number of branches (Fig. 2Ba) and size of the branched area (Fig. 2Bb), and the reduced distance between branches (Fig. 2Bc). In addition, there is an increased number of TEBs, the highly proliferative structures that are responsible for generating the ductal epithelial tree at their distal ends, suggesting enhanced proliferation in pubertal and young adult *Ackr2*^−/−^ mice (Fig. 2Bd). Finally, *Ackr2*^−/−^ mice displayed significantly increased ductal elongation at 8 weeks (Fig. 2Be). By 12 weeks TEBS have regressed, the ductal tree has reached the end of the fat pad and ductal outgrowth is completed, and at this stage there is no difference between wild-type and *Ackr2*^−/−^ animals (Fig. 2). ACKR2 does not affect the morphology of individual TEBs or the thickness of epithelial branches throughout development (Fig. S1A,B), nor were any differences in inguinal lymph node size noted (Fig. S1C). Thus, ACKR2 deletion is associated with accelerated mammary gland development in late puberty.

### ACKR2 is expressed by stromal cells in the mammary gland

**Fig. 3.**
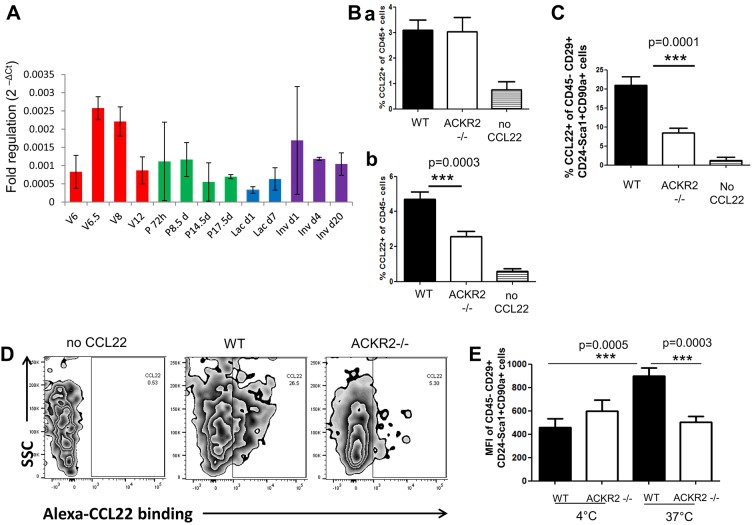
**ACKR2 is expressed by stromal cells in the mammary gland.** (A) Relative levels of ACKR2 expression (2^−ΔCT^) during virgin mammary gland development (*n*=3), pregnancy, lactation and involution (*n*=2). (B) Flow cytometry was used to determine the percentage of uptake of the fluorescent ACKR2 ligand CCL22 by (1) CD45^+^ and (2) CD45^−^ wild-type and *Ackr2^−/−^* mammary gland cells. Results were combined from three individual experiments (wild type, *n*=16; *Ackr2^−/−^*, *n*=14). (C) Alexa-CCL22 uptake by a stromal population of CD45^−^CD29^+^CD24^−^Sca1^+^CD90^+^ cells in wild-type and *Ackr2^−/−^* mice. Results were combined from two individual experiments (wild type, *n*=10, *Ackr2^−/−^ n*=10). (D) Representative FACS plots of Alexa-CCL22 uptake by CD45^−^CD29^+^CD24^−^Sca1^+^CD90^+^ cells in wild-type and *Ackr2^−/−^* mice. Alexa-CCL22+ populations were gated using control cells without fluorescent CCL22 added. (E) Mean fluorescence intensity of Alexa-CCL22 indicates binding and uptake by CD45^−^, CD29^+^,CD24^−^Sca1^+^,CD90^+^ fibroblasts at 4°C and 37°C. Results were combined from two individual experiments (wild type, *n*=10, *Ackr2^−/−^ n*=10). Significantly different results are indicated. Error bars represent s.e.m.

ACKR2 expression was determined by qRT-PCR arrays and shown to be expressed in the mammary gland at each of the developmental stages investigated (Fig. 1A and Fig. 3A). Maximal expression of ACKR2 in virgin mice was seen between weeks 6.5 and 8. More modest expression was seen throughout pregnancy and during lactation and involution. ACKR2 expression therefore coincides with the time window in which we see accelerated mammary gland development in the *Ackr2*^−/−^ mice. To determine which resident mammary gland cells express ACKR2, inguinal mammary gland tissue was enzymatically digested from wild-type and *Ackr2*^−/−^ mice and analysed by flow cytometry for uptake of the fluorescently labelled ACKR2 ligand CCL22 (Alexa-CCL22). There was no difference in Alexa-CCL22 uptake by wild-type and *Ackr2*^−/−^ CD45^+^ cells, indicating that ACKR2 is not expressed by leukocytes in the mammary gland (Fig. 3Ba). However, Alexa-CCL22 uptake by CD45^−^ cells was lower in *Ackr2*^−/−^ animals (Fig. 3Bb). Specifically, Alexa-CCL22 uptake was markedly reduced in a population of fibroblastic cells (Singhal et al., 2016), which are CD45^−^ CD24^−^ CD29^+^ Sca1^+^ CD90^+^, indicating that ACKR2 is expressed by this stromal population (Fig. 3C,D). Pre-adipocytes are an important component of the mammary stroma that are CD24^−^ CD29^+^ Sca1^+^ CD34^+^ (Berry and Rodeheffer, 2013); however, the Alexa-CCL22-binding cells are CD34^−^ and do not belong to this population. We next analysed the ability of the ACKR2 on these cells to bind and/or internalise ligand. As shown in Fig. 3E, and in keeping with the predominantly intracellular localisation of ACKR2 (Blackburn et al., 2004), little ligand binding was detected at 4°C, at which temperature ligand internalisation will be minimal. By contrast, ACKR2-dependent internalisation of ligand was seen at 37°C. Together, these data indicate that ACKR2 is expressed on a stromal fibroblastic population in the mammary gland and that, on these cells, it is capable of both binding and internalising ligand.

### ACKR2 regulates the levels of inflammatory CC-chemokines in the mammary gland

**Fig. 4.**
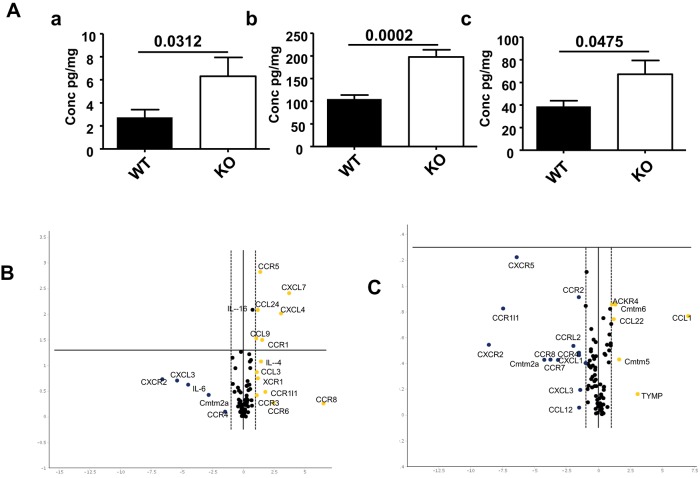
**Chemokine levels** **in *Ackr2^−/−^* mammary glands.** (A) Multiplex measurement of protein concentration of (a) CCL7; (b) CCL11 and (c) CCL12 in whole mammary gland homogenates. *n*=8 per group. (B,C) Volcano plots of fold change versus significance between wild-type and *Ackr2^−/−^* glands at (B) 6.5 weeks and (C) 8 weeks. Log_2_ fold change in gene expression between wild-type and *Ackr2^−/−^* mammary glands are plotted against *t*-test *P* values. Thresholds for twofold change are indicated by vertical lines; significance (*P*<0.05) is indicated by a horizontal line; *n*=3 per group.

One likely mechanism to explain the involvement of ACKR2 in regulating mammary gland development relates to its ability to limit inflammatory CC-chemokine levels in tissues. We therefore used multiplexing approaches to assess chemokine levels in the mammary gland, focusing on the 6.5 week time point. Many chemokines were undetectable at this time point and others were detectable but displayed no differences in protein levels between wild-type and *Ackr2*^−/−^ mammary glands (Fig. S2). In addition, no differences in expression of the macrophage differentiation factor CSF1 were seen (Fig. S2). However, elevated levels of CCL7, CCL11 and CCL12 were seen in *Ackr2*^−/−^ mammary glands (Fig. 4A). Notably, qRT-PCR-based array analysis comparing the transcriptional profiles of wild-type and *Ackr2*^−/−^ mammary glands in 6.5- (Fig. 4B) and 8-week-old mice (Fig. 4C) did not detect differences in the transcript levels of these three chemokines (Table S1). Thus, their elevated levels are a result of deficient scavenging in *Ackr2*^−/−^ mice and not increased transcription.

### ACKR2 controls the accumulation of macrophages in the mammary gland

**Fig. 5.**
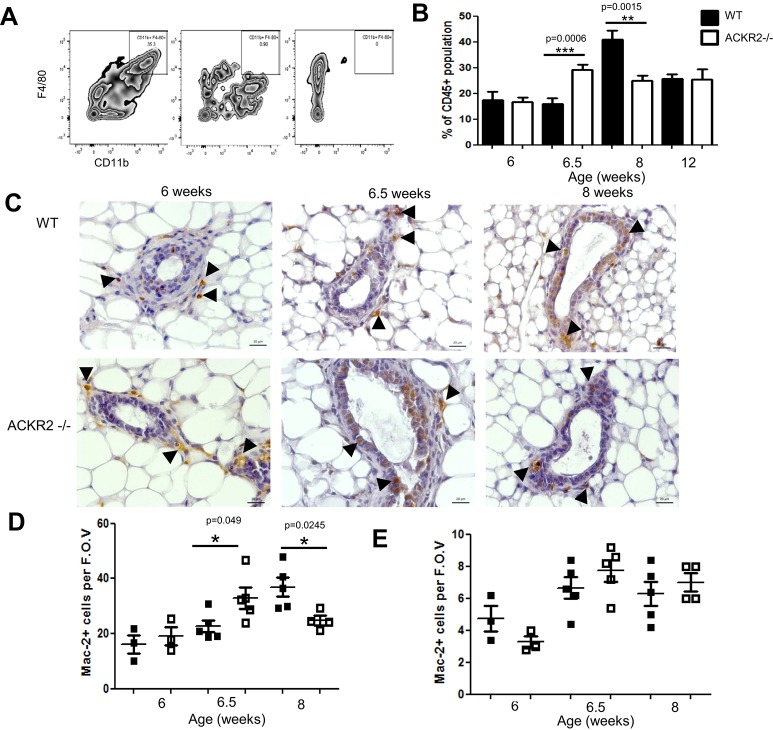
**ACKR2 controls the accumulation of macrophages in the mammary gland.** (A) Flow cytometry was used to identify the percentage of CD11b^+^ F4/80^+^ macrophages within the CD45^+^ population of the mammary gland during development. Gating based on fluorescence minus one (FMO) controls is shown. (B) FACS analysis of wild-type and *Ackr2^−/−^*-deficient glands was carried out at 6, 6.5, 8 and 12 weeks. Results were combined from two independent experiments, *n*=8-11. (C) Mac2+ cells, indicated by arrowheads, were visualised within the developing mammary gland by immunohistochemistry. Representative 40× bright-field images of Mac2-stained sections from wild-type and *Ackr2^−/−^* glands are shown. (D,E) The number of Mac2+ cells per gland was the average counted from five individual FOV (40×) either (D) around TEBs or (E) from within adipose tissue, from 6 week (*n*=3), 6.5 week (*n*=5) and 8 week (wild type *n*=5, KO *n*=4) glands. Significantly different results are indicated. Error bars represent s.e.m.

The three chemokines identified as being increased at the protein level in *Ackr2*^−/−^ mammary glands are regulators of macrophage, eosinophil and mast cell recruitment that act in differing combinations through chemokine receptors CCR1, CCR2, CCR3 and CCR5 (White et al., 2013). Importantly, ACKR2 deficiency does not appear to alter eosinophil or mast cell recruitment, both of which are known to mediate effects on branching morphogenesis (Gouon-Evans et al., 2000; Lilla and Werb, 2010) (Fig. S3). By contrast, differences in macrophage dynamics were observed in *Ackr2*^−/−^, compared with wild-type, mammary glands following FACS analysis of enzymatically digested mammary gland cells from pubertal and adult mice. There was no difference in the percentage of CD45^+^ CD11b^+^ F4/80^+^ macrophages (Fig. 5A) isolated from wild-type and *Ackr2*^−/−^ glands at 6 weeks; however, at 6.5 weeks the percentage of macrophages in *Ackr2*^−/−^ glands was significantly higher than in wild-type (Fig. 5B). By 8 weeks this differential had reversed. In addition, Mac2-stained sections from mice at 6.5 weeks indicated higher numbers of macrophages associated with the TEBs in *Ackr2*^−/−^ compared with wild-type mice, whereas the reverse was observed at 8 weeks (Fig. 5C). This was quantified (Fig. 5D), which confirmed the altered numbers of TEB-associated macrophages in the *Ackr2*^−/−^ mice were statistically significant. Importantly, no differences were seen in the numbers of macrophages in the adipose tissues surrounding the TEBs (Fig. 5E). This accelerated recruitment of macrophages in *Ackr2*^−/−^ mice is consistent with the precocious developmental phenotype observed. In adult 12-week-old mice there is no difference in the number of macrophages, consistent with branching being unaffected at this time point (Figs 2 and 5B).

### CCR2 is not the sole regulator of macrophage recruitment and dynamics in the developing mammary gland

**Fig. 6.**
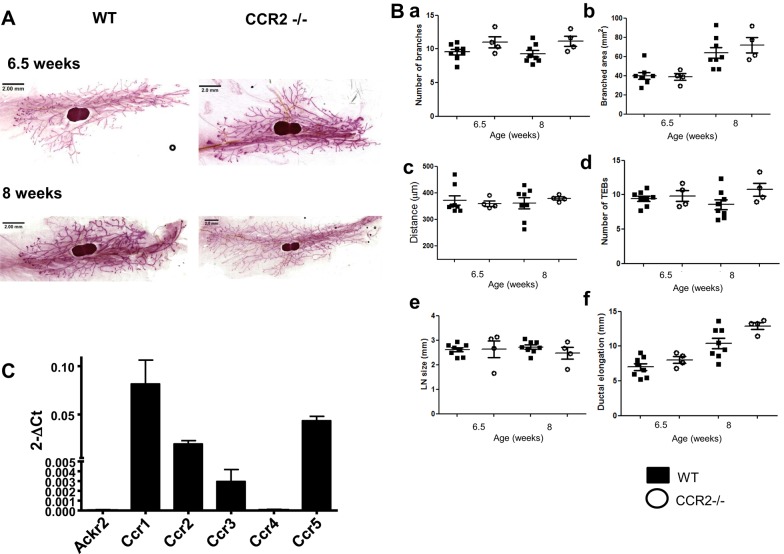
**CCR2 does** **not control branching morphogenesis within the developing mammary gland.** (A) Representative Carmine Alum staining of wild-type and *Ccr2^−/−^* mammary gland whole mounts at 6.5 and 8 weeks of age is shown. (B) *Ccr2^−/−^* stained whole mounts were quantified for: (a) the number of branches; (b) the branched area; (c) the distance between branches; (d) the number of TEBs; (e) lymph node size; and (f) ductal elongation (*n*=4). Each measurement was from three FOV (5×) per gland. Wild-type data points are the same as those used in Fig. 2B. Results were not statistically significant. (C) qRT-PCR analysis of expression of ACKR2, CCR1, CCR2, CCR3, CCR4 and CCR5 in sorted mammary gland macrophages. Error bars represent s.e.m.

In embryonic skin, ACKR2 regulates the gradient of the CCR2 ligand CCL2 to control macrophage proximity to the developing lymphatic network; as such, *Ccr2^−/−^* mice have a reciprocal phenotype to *Ackr2*^−/−^ mice, including reduced lymphatic vessel density (Lee et al., 2014). To determine whether mammary gland branching morphogenesis is impaired in the absence of CCR2 during puberty, Carmine Alum whole-mount staining of age- and weight-matched virgin wild-type and *Ccr2^−/−^* glands was carried out. The number of branches, the branched area and the distance between branches was unaffected by CCR2 deletion, indicating that ACKR2 controls branching morphogenesis in the mammary gland through a distinct mechanism that is independent of CCR2 (Fig. 6A,Ba-c). There were also no differences in the numbers of TEBs, in LN size or in ductal elongation in the *Ccr2^−/−^* mice (Fig. 6Bd-f). Of note, CCR2 does affect morphology, as the size of individual TEBs are increased in *Ccr2^−/−^* mice at 6.5 weeks (Fig. S4A), and *Ccr2^−/−^* branches are marginally thinner at 8 weeks (Fig. S4B). Our data further indicate that *Ccr2^−/−^* mice display no differences in macrophage, eosinophil or mast cell recruitment (Fig. S5).

These data therefore raise the issue of the identity of the chemokine receptor(s) responsible for the exaggerated macrophage recruitment in *Ackr2^−/−^* mammary glands. qRT-PCR analysis of wild-type macrophages purified from the mammary gland at week 8 demonstrate that although these cells did not express ACKR2, they simultaneously expressed CCR1, CCR2 and CCR5, and weakly expressed CCR3 (Fig. 6C). It is therefore likely that these macrophages are responsive to a range of chemokine receptor ligands. Unfortunately, mouse models allowing an analysis of the impact of compound receptor deficiency are not currently available and thus the precise receptor use involved in macrophage recruitment to the developing mammary gland remains to be defined.

## DISCUSSION

The importance of immune cells in mammary gland development is well known (Gouon-Evans et al., 2000; Lilla and Werb, 2010); however, the molecular cues guiding their migration into, and positioning within, the gland are not well understood. Indeed, relatively little is known about the roles of chemokines and their receptors in normal mammary gland development. In this study we have demonstrated that the atypical chemokine receptor ACKR2 is expressed by a fibroblastic population of stromal cells and is a key regulator of macrophage recruitment and positioning in the murine mammary gland. Communication between the stroma and epithelium is crucial, as the stroma provides a wealth of growth factors, ECM proteins and microRNAs that tightly regulate ductal elongation and patterning, the disruption of which often leads to aberrant development (Kayo et al., 2014; Parmar and Cunha, 2004). Here, we add a further example of crosstalk between the stroma and epithelium, and the immunological signals that determine the degree of branching and spacing between ducts.

*Ackr2^−/−^* mice exhibit rapid mammary gland development throughout late puberty, which is characterised by increased numbers of TEBs and branches, and is initiated by earlier recruitment of macrophages to the mammary gland. Specifically, the onset of accelerated development and macrophage recruitment occurs at 6.5 weeks in *Ackr2^−/−^* mice, when ACKR2 expression peaks in wild-type mice. This is associated with increased level of macrophage chemoattractants in the *Ackr2^−/−^* mammary gland and suggests that ACKR2 controls the temporal onset of mammary gland development by regulating chemokine availability. Together, our data indicate that 6.5 weeks is a key developmental time point at which ACKR2 limits macrophage recruitment. Developmentally, this coincides with the onset of sexual maturity (Topper and Freeman, 1980). Overall, mammary gland development in *Ackr2^−/−^* mice occurs ∼2 weeks earlier than in wild-type mice. Given that the average lifespan of a mouse in the wild is approximately 6 months, this represents a potentially important acceleration of this specific aspect of sexual maturity.

Notably, postnatal control of macrophage recruitment by ACKR2 occurs through a distinct mechanism to that described in embryonic skin, which is dependent on CCL2 and its receptor CCR2. In contrast to embryonic skin, *Ccr2^−/−^* mice do not exhibit an inverse phenotype in the mammary gland, as epithelial branching is not affected. Furthermore, transcriptional analysis of pubertal mammary glands revealed that CCL2 and CCR2 are unchanged in *Ackr2^−/−^* mice. However, CCR2 does have effects on the morphology of the ductal tree, with larger TEBs in pubertal mice and thinner branches in adults. These effects are not caused by altered macrophage, eosinophil or mast cell recruitment. qRT-PCR analysis of mammary gland macrophages demonstrated expression of CCR1, CCR2 and CCR5, as well as low level expression of CCR3. The known complex and potentially redundant interactions between inflammatory CC-chemokines and their receptors suggests that these cells could use a variety of chemokines and cognate receptors for migration into the mammary gland. The key chemokine receptors occupy a tight chromosomal locus (Nomiyama et al., 2011); therefore, compound chemokine receptor-null mice, which would directly address this issue are not available at present, and thus the precise receptors used by macrophages during recruitment to the mammary gland await further characterisation. Notably, Fig. 4 shows increased transcription of the inflammatory chemokine receptors CCR1 and CCR5 in *Ackr2^−/−^* mice at 6.5 weeks, suggesting, again, that combinations of these receptors may be involved in macrophage recruitment.

Here, we provide, for the first time, a comprehensive analysis of chemokine transcription to gain broad insights into their roles throughout the full course of virgin and reproductive development. It has been shown that CCL11 (eotaxin)-mediated recruitment of eosinophils is required for proper TEB formation and ductal elongation (Gouon-Evans et al., 2000). Here, we observed that expression of CCL11 is highest in developing virgin and involuting glands, suggesting recruitment of eosinophils is reduced during pregnancy and lactation. In a previous study, CCL28 and CCR10 have been shown to facilitate the homing and accumulation of IgA antibody-secreting cells during lactation (Morteau et al., 2008). Consistent with this, we observed high levels of CCL28 during lactation. In addition, CXCL1 has been previously reported to comprise part of an involution-associated gene signature on day 1 of involution, which is again identified in this study and suggests that neutrophil influx is important during involution (Clarkson et al., 2003; Reed and Schwertfeger, 2010; Stein et al., 2004). CXCL14 (BRAK) was initially identified in breast tissue and is expressed here throughout mammary development, with the highest level present during involution (Hromas et al., 1999). CXCR4 is known to be expressed at low levels in normal breast tissue, but is characteristic of malignant epithelial cell growth, and is important for metastasis (Balkwill, 2004; Wiseman and Werb, 2002). In this study, CXCR4 is strongly associated with pregnancy, which could have implications for our understanding of pregnancy-associated breast cancer risk. Similarly CXCL12, a ligand of both CXCR4 and ACKR3, is associated with advanced breast cancer in humans, and is expressed at high levels during puberty and pregnancy in our study (Fridrichova et al., 2015) (data not shown). ACKR3 (CXCR7) promotes breast cancer cell proliferation (Salazar et al., 2014) and is upregulated here during lactation and involution (data not shown).

Understanding the molecular basis of mammary gland development is crucial to help us understand how breast cancers arise. Normal developmental processes which involve proliferation of the epithelium have high tumorigenic potential, for example the ability to reinitiate cellular proliferation throughout reproduction, produce anti-apoptotic signals during lactation to prevent premature involution, and to induce angiogenesis during frequent vasculature remodelling. In addition, the highly proliferative nature of the TEB and its ability to invade stromal tissues strongly resemble features of some solid tumours (Wiseman and Werb, 2002). It is also important to further understand how macrophages are regulated within the mammary gland, as they are vital both for pubertal development and metastatic tumour progression (Gouon-Evans et al., 2000; Lin et al., 2001). Here, we describe an important role for ACKR2 in the control of macrophages that potentiate epithelial branching during normal breast development. This may provide insights into the origin and development of tumours and potentially allow therapeutic intervention.

## MATERIALS AND METHODS

### Animals

Animal experiments were carried out using female age-matched mice and conformed to the animal care and welfare protocols approved by the University of Glasgow and carried out under the auspices of a UK Home Office Project Licence. C57BL/6 mice, *Ackr2^−/−^* mice (Jamieson et al., 2005) and *Ccr2*^−/−^ mice were bred in-house at the specific pathogen-free facilities at the Beatson Institute for Cancer Research (Glasgow, UK).

### Transcriptional analysis

To isolate RNA, the inguinal lymph node (LN) was removed and mammary tissue from the fourth inguinal gland was minced coarsely with dissecting scissors before being added to Qiazol with 7 mm stainless steel beads (Qiagen). Samples were processed using a Tissuelyser LT for 10 min at a rate of 50 s^−1^, and the miRNeasy kit as described in the manufacturer's instructions (Qiagen). RNA samples from reproductive stages were collected during pregnancy, lactation and forced involution, as described previously (Olijnyk et al., 2014; Stein et al., 2004). Purified mammary gland macrophages were lysed in Buffer RLT and processed using a QIAshredder (Qiagen) before using a microRNeasy kit (Qiagen) to isolate RNA. From tissue samples, 400 ng RNA was converted to cDNA using the RT^2^ First Strand kit and 1-2 ng RNA from purified macrophage samples were preamplified using the RT2 PreAMP cDNA Synthesis Kit and array-specific primers (Qiagen). Transcription levels were determined by quantitative real-time polymerase chain reaction (qRT-PCR) using the mouse Chemokine and Chemokine Receptor RT^2^ profiler PCR array (Qiagen) and RT^2^ SYBR Green ROX qPCR Mastermix (Qiagen), as described in the manufacturer's instructions. Fold regulation was determined using the 2^(−ΔCt)^ method, where ΔC_T_ is calculated as C_T target_−C_T normaliser_. Normalisation was carried out using the arithmetic mean of the C_T_ of GAPDH, Hsp90a and β-actin. Volcano plots were used to identify significant gene expression changes. Non-supervised hierarchical clustering was used to show common gene expression and co-regulated genes across groups using the SABiosciences data analysis software (Qiagen).

### Whole-mount analyses

Fourth inguinal mammary glands were spread onto Superfrost Plus microscope slides (Thermo Scientific) and fixed overnight in 10% neutral buffered formalin (NBF) (Leica) at 4°C. Tissue was dehydrated by incubation for 1 h in distilled water, followed by 70% ethanol and 100% ethanol (VWR international) before overnight incubation in xylene (VWR international) at room temperature. Tissues were then rehydrated by subsequent 1 h incubations in 100% ethanol, 70% ethanol and distilled water, and stained in Carmine Alum solution [0.2% (w/v) carmine and 10 mM aluminium potassium sulphate (Sigma)], overnight at room temperature. Tissue was dehydrated again by 1 h incubations in distilled water, 70% ethanol and 100% ethanol, and overnight incubation in xylene at room temperature. Finally, glands were mounted using DPX (Leica) and stitched bright-field images at 10× magnification were obtained using the Evos Cell Imaging System (Thermo Scientific). Ductal elongation, post-LN branched area and LN size were measured using ImageJ 1.48v (Schneider et al., 2012). Bright-field images (5×) were obtained using the Zeiss Axioimager M2 with Zen 2012 (blue edition) software. The numbers of branches and TEBs were counted as the average from three individual fields of view (FOV) from each whole mount. The average distance between branches and branch thickness was calculated from three measurements from each of 3 individual FOV.

### Immunocytochemical analyses

Fourth inguinal mammary glands were fixed in 10% NBF, treated using a tissue processor (Thermo Scientific) and embedded in paraffin wax using an embedding suite (Thermo Scientific). Sections were cut at 4-6 μm on a microtome (Thermo Scientific), floated in a 40°C water bath (Thermo Scientific) and collected onto Superfrost Plus microscope slides before being baked at 60°C for 30 min. After treatment with xylene for 10 min, slides were rehydrated by 5 min incubations in 100% ethanol, 70% ethanol and running water. After this, they were placed in distilled water and rinsed with PBS (Sigma). H_2_O_2_ (3% ; Fisher Scientific) was applied for 5 min before being rinsed again with PBS. Slides were blocked using a 20% goat serum (Vector Labs) before staining overnight at 4°C with a 1 in 6000 dilution in PBS containing 1% BSA (Sigma) of either primary rat anti-mouse Mac2 antibody (Cedarlane) or the isotype control antibody IgG2a (BD Biosciences). Slides were washed three times in PBS for 5 min before adding a 1 in 200 dilution of goat anti-rat IgG (Vector Labs). Slides were washed in PBS and a 1 in 200 dilution of Extravadin-peroxidase (Sigma) was applied for 30 min before being washed again. Diaminobenzidine (DAB) substrate (Vector Labs) was applied and quenched with tap water. Finally, Haematoxylin was added as a counterstain before being dehydrated in 70%, 90% and 100% ethanol and xylene (VWR International), and mounted in DPX.

### Mammary gland cell isolation

Inguinal lymph node was removed and mammary tissue was coarsely minced before enzymatic digestion with 3 mg/ml (w/v) collagenase type 1 (Sigma) and 1.5 mg/ml (w/v) trypsin (Sigma) in serum-free Leibovitz L-15 medium (Sigma) in a shaking incubator at 200 rpm (37°C for 45 min). The suspension was shaken by hand for 15 s before addition of an equal volume of ice-cold Leibovitz L-15 medium supplemented with 10% fetal calf serum (Invitrogen) (L-15/10% FCS) and centrifugation for 5 min at 350 ***g***. Supernatants were gently aspirated and erythrocytes were lysed using Red Blood Cell Lysing Buffer Hybri-Max (Sigma) for 1 min and washed in PBS. Cells were washed again in PBS with 5 mM EDTA, resuspended in 2 ml 0.25% Trypsin-EDTA (Sigma) and incubated at 37°C for 2 min before addition of 5 ml of serum-free L15 containing 1 μg/ml DNase1 (Sigma) for 5 min at 37°C. An equal volume of L-15/10% FCS was added to stop the reaction and cells were filtered through a 40 μm cell strainer before a final wash in FACS buffer, PBS containing 1% FCS and 5 mM EDTA. Mammary gland macrophages were isolated using MACS LS columns (Miltenyi Biotec) by staining with F4/80 conjugated with APC and anti-APC microbeads.

### Proteomic analysis

Mammary tissue without the inguinal lymph node was coarsely minced before being frozen in liquid nitrogen, crushed with a mortar and pestle, and resuspended in dH_2_O containing protease inhibitors (Pierce). Protein levels were determined using a customised Magnetic Luminex Multiplex assay (R&D Systems), as described in the manufacturers' instructions, and read using a Bio-Rad Luminex-100 machine. Protein concentration of tissue samples was determined using the BCA assay (Pierce).

### Flow cytometry

The following antibodies were obtained from BioLegend and used at a dilution of 1:200: CD45 (30-F11), CD11c (N418), CD11b (M1/70), CD117 (2B8), CD24 (M1/69), CD29 (HMβ1-1) and CD90.2 (30-H12). F4/80 (BM8) from eBiosciences and Siglec F (E50-2440) from BD Biosciences were used at a dilution of 1:200. Sca1 (D7) from Miltenyi biotec was used at a dilution of 1:50. Dead cells were excluded using DRAQ7 (BioStatus) or Fixable Viability Dye eFluor 506 (ebioscience). Macrophages were defined as CD11b^+^ F4/80^+^, eosinophils as CD11c^−^ Siglec F^+^ and mast cells as CD117^+^. Fluorescent chemokine uptake experiments were carried out using 0.5 μg AF647-labelled human CCL22 (Almac, Scotland UK) as described previously (Ford et al., 2013). FACS was performed using an LSRII, (BD Biosciences) and analysed using FlowJo V10.

### Statistical analysis

Data were analysed using GraphPad Prism 5.03. *F* tests were carried out to compare variances and normality was assessed using the Kolmogorov–Smirnov test. Two-tailed, unpaired *t*-tests were used to analyse data with normal distribution, Mann–Whitney or one-way ANOVA with Tukey's multiple comparison tests was applied for comparison of groups, as appropriate. Significance was indicated by *P<0.05. Error bars indicate s.e.m.
